# 2D Raman band splitting in graphene: Charge screening and lifting of the *K*-point Kohn anomaly

**DOI:** 10.1038/s41598-017-13769-3

**Published:** 2017-10-19

**Authors:** Xuanye Wang, Jason W. Christopher, Anna K. Swan

**Affiliations:** 1 Department of Electrical and Computer Engineering, Photonics Center, Boston University, 8 St Mary’s Street, Boston Massachusetts, 02215 United States of America; 2 Department of Physics, Boston University, 590 Commonwealth Avenue, Boston, MA 02215 United States of America

## Abstract

Pristine graphene encapsulated in hexagonal boron nitride has transport properties rivalling suspended graphene, while being protected from contamination and mechanical damage. For high quality devices, it is important to avoid and monitor accidental doping and charge fluctuations. The 2D Raman double peak in intrinsic graphene can be used to optically determine charge density, with decreasing peak split corresponding to increasing charge density. We find strong correlations between the *2D*_1_ and 2*D*_2_ split vs 2D line widths, intensities, and peak positions. Charge density fluctuations can be measured with orders of magnitude higher precision than previously accomplished using the G-band shift with charge. The two 2D intrinsic peaks can be associated with the “inner” and “outer” Raman scattering processes, with the counterintuitive assignment of the phonon closer to the *K* point in the *KM* direction (outer process) as the higher energy peak. Even low charge screening lifts the phonon Kohn anomaly near the *K* point for graphene encapsulated in hBN, and shifts the dominant intensity from the lower to the higher energy peak.

## Introduction

Raman spectroscopy is a versatile experimental technique for exploring fundamental properties of graphene and characterizing properties such as layer thickness, defect density, charge and strain^1^. The 2D Raman mode is of particular interest, as it involves two D band phonons with opposite, non-zero momenta. Despite the higher order process and non-zero momentum phonons, the 2D mode is the strongest Raman mode in single layer graphene. The double resonance (DR) mechanism^2^ explains why this higher order phonon mode is so strong; resonant virtual electron and hole scattering picks phonon *q* vectors that satisfy momentum and energy conservation. The DR process together with the strong *K*-point Kohn anomaly is why the 2D line provides so much information about the electronic and phonon dispersions around the *K* and *K* **’** points. The phonon Kohn anomaly near *K* softens the D band phonon energies conically near *K*, resulting in a discontinuity of the derivative of the dispersion at *K* ^3^. The slope of the phonon dispersion is a direct measure of the electron-phonon coupling (EPC) strength^3^. The DR and phonon dispersion are responsible for the shift in the 2D band energy with changing laser excitation energy^4^. Similarly, the strong dependence of the 2D mode on dielectric screening is readily observable by the shift in the 2D position for different environments, e.g., suspended graphene, graphene on SiO_2_, graphene on top of hexagonal boron nitride (hBN) or encapsulated in hBN, etc^5^. Larger dielectric screening^6^ will reduce the Fermi velocity, *v*_*F*_, so that the DR conditions will select larger phonon energy. The screening affects *v*_*F*_ since electron energies are much higher than expected from a single particle picture, with a significant self-energy correction in a 2D material, in particular for free standing graphene near the charge neutrality point (CNP)^7–9^. The self-energy is decreasing as ε^−1^, where ε is the effective dielectric constant of the environment^6^. Charging the graphene layer will also renormalize and reduce the self-energy and therefore *v*_*F*_. For example, a study of gated graphene on hexagonal boron nitride (hBN) examined with scanning tunnelling spectroscopy demonstrated a change of *v*_*F*_ from 1.3 to 1.05 × 10^6^ m/s as the doping increased one order of magnitude from 2 × 10^11^ cm^−2^ to 2 × 10^12^ cm^−2^ ^9^. Ultraclean suspended graphene with mobility reaching 10^6^ cm^2^/Vs have shown *v*_*F*_ reaching 3 × 10^6^ m/s, measured by Shubnikov-de Haas oscillations^7^. Screening will also have an effect on the phonon dispersion, as it may decrease the strength of the EPC, and thereby weakening the Kohn anomaly at *K*, resulting in higher phonon energies with increased screening^5,10,11^. The reduced *v*_*F*_ and reduced Kohn anomaly with screening are both expected to lead to a higher energy 2D peak. Hence, changes to the dispersion of the Dirac cone and the phonon branch can be experimentally deduced from the 2D peaks.

**Figure 1:**
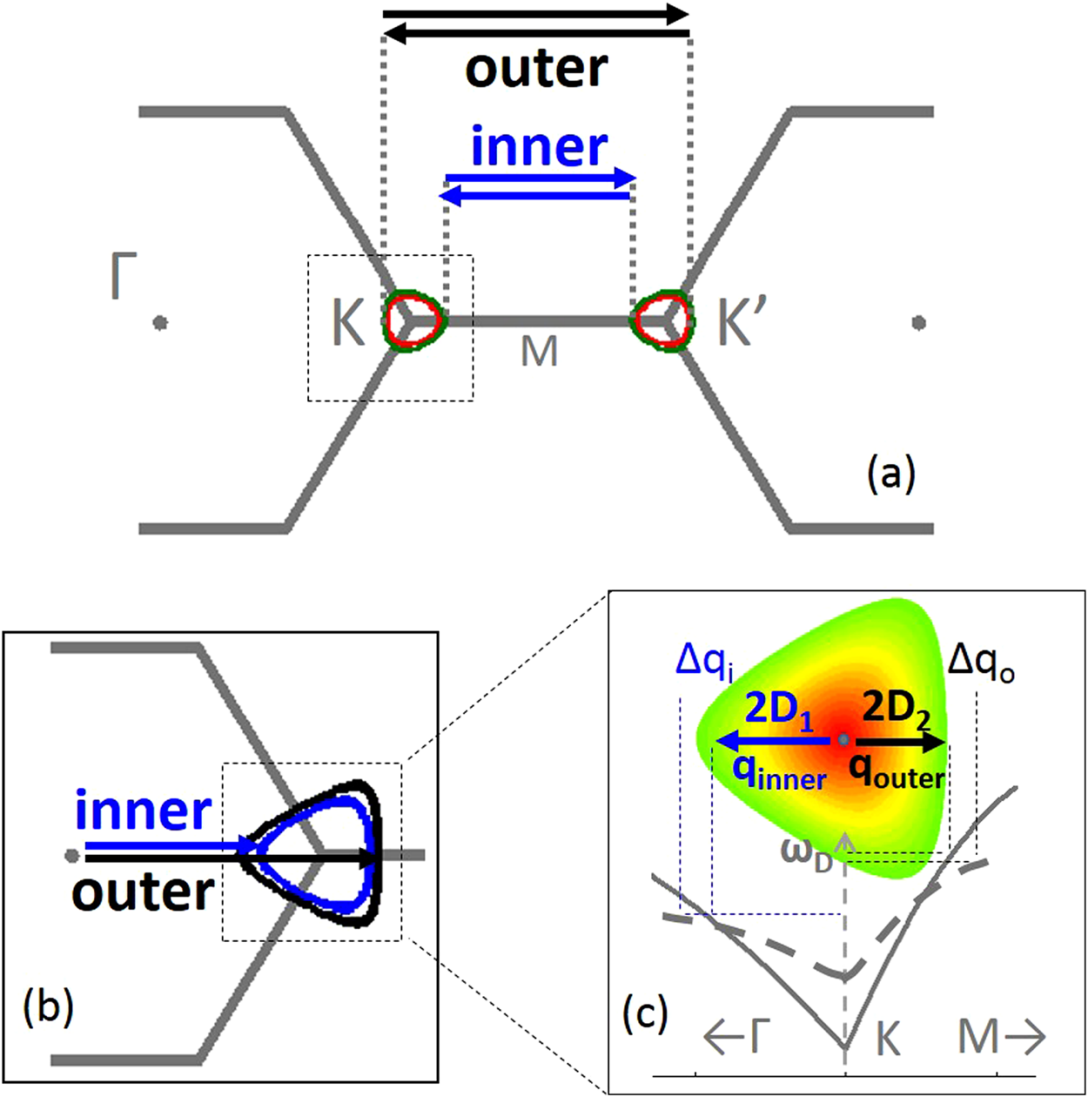
Schematic of the double resonance mechanism. (**a**) Top view of the *K* and *K’* electron dispersion iso-energy contours for the selected *k* vectors. Red and green circles in (**a**) are the iso-energy contours *E*_*L*_ */2 and* (*E*_*L*_ */2 − hcω*_*D*_) at *K* and *K’*, respectively for the red (633 nm) and green laser (532 nm) excitation wavelengths. The black and blue arrows show the resonant *k*-vectors for the outer and inner processes, respectively. These vectors pick out the phonon iso-energy contours shown in (**b**) and the phonon *q* vectors in (**c**) shown both in a top view (top) and a crosscut (bottom). If the phonon dispersion does not fully match the electronic dispersion, there will be an energy difference between the inner and outer phonon, here shown as the outer resonance having higher energy. The dashed line in the cross cut qualitatively depicts the change in phonon dispersion with increased screening. The *Δq* addition to the *q* vectors represents the added length with increased screening due to the change in Fermi velocity and therefor the electronic iso-energy contours.

The DR method can be used for mapping out a contour of participating phonon wave-vectors^12,13^. The vectors are found by translating the iso-electronic energy contour determined by half the laser energy at *K*, with a vector that puts the *K* iso-electronic energy contour tangentially in contact with the slightly smaller iso-energy contour (*E*_*L*_ */2 − hcω*_*D*_) at *K’*, shown in Fig. 1(a). From this argument, it is clear that the trigonal warping of the Dirac cone determines the *q* vectors associated with the 2D Raman peak. While the DR Raman scattering is a two-dimensional process, the strongest contributions come from the high symmetry direction *ΓK* and *KM*, so that we can consider the one-dimensional case where electron (hole) scattering from *ΓK* to *K’Γ* followed by hole (electron) scattering back from *K’Γ* to *ΓK* are referred to as the *outer process* and the similar process between *KM* and *MK’* as the *inner process* ^11–13^, as shown in Fig. 1(a). Because of the trigonal warping of the Dirac cone, there is a difference between the length of the selected inner and outer vectors, shown in Fig. 1(a–c). If the phonon dispersion around *K* was cylindrically symmetric, the 2D peak would yield two very different phonon frequencies from the inner and outer process since *q*_*i*_ > *q*_*o*_ where *q* is measured from the *K* point with cylindrical coordinates, shown in Fig. 1(c). However, the phonon dispersion has an opposite trigonal warping so as to almost perfectly compensate for the electronic distortion^12^. Indeed, early in the study of graphene, a symmetric 2D peak for graphene on SiO_2_/Si was identified as a signature of a single layer^14^. Later studies showed that the intrinsic 2D line shape of single layer suspended graphene is asymmetric^15–17^, but reverts to a symmetric line shape when charge is added by field doping to 2 × 10^11^ cm^−2^, indistinguishable in shape from graphene on SiO_2_^18^. Hence, we can deduce that there is not a perfect compensation of the phonon trigonal warping at low doping condition, and that the two peaks can reveal the behaviour of the inner and outer scattering processes, respectively. In addition, the energy upshift of the 2D peak is indicative of screening due to changes in the Fermi velocity, which has been pointed out previously^19,20^, but also due to changes in the phonon velocities^12^, addressed here.

Here we are considering the effect of very low doping levels in ultra-clean graphene, dielectrically screened by encapsulation in hBN. Encapsulated graphene possesses many of the superior transport properties of suspended graphene with the added advantage of being mechanically more robust and easier to fabricate^21^. The accidental doping in these samples is approximately ~10^11^ cm^−2^ or less as deduced from transport measurements^22^, more than an order of magnitude lower than for graphene on SiO_2_. We find strong correlations between the 2D peak splitting and the relative peak intensities and linewidths. The *decrease* of the *2D*_*2*_ peak energy with charge screening is opposite to expectations. It follows from a crossover, shown in Fig. 1(c) bottom, of the phonon dispersions for unscreened and screened graphene, as have been predicted^10,11^. This is a consequence of screening the Kohn anomaly at *K*. One has to carefully consider the ratio of the inner and outer Fermi velocities with the ratio of the inner and outer phonon velocities before determining if *2D*_1_ is an inner or outer process. Here we present evidence to suggest that the lower energy *2D*_1_ peak originates from the inner process, based on the calculated stronger intensity from the inner process in the low doping regime^12^, and linewidth behaviour. The analysis below is based on the assumption that increasing the charge also increases the screening. This assumption is supported by the reduction in Fermi velocity with increasing charge level, especially close to the charge neutrality point, attributed to screening the self-energy correction of the electronic energy dispersion^7,9^.

On a practical level, the peak split between *2D*_1_ and 2*D*_*2*_ can be used as a measure of charge doping two orders of magnitude lower than is possible to measure using the G band response to doping^23,24^.

## Results

**Figure 2:**
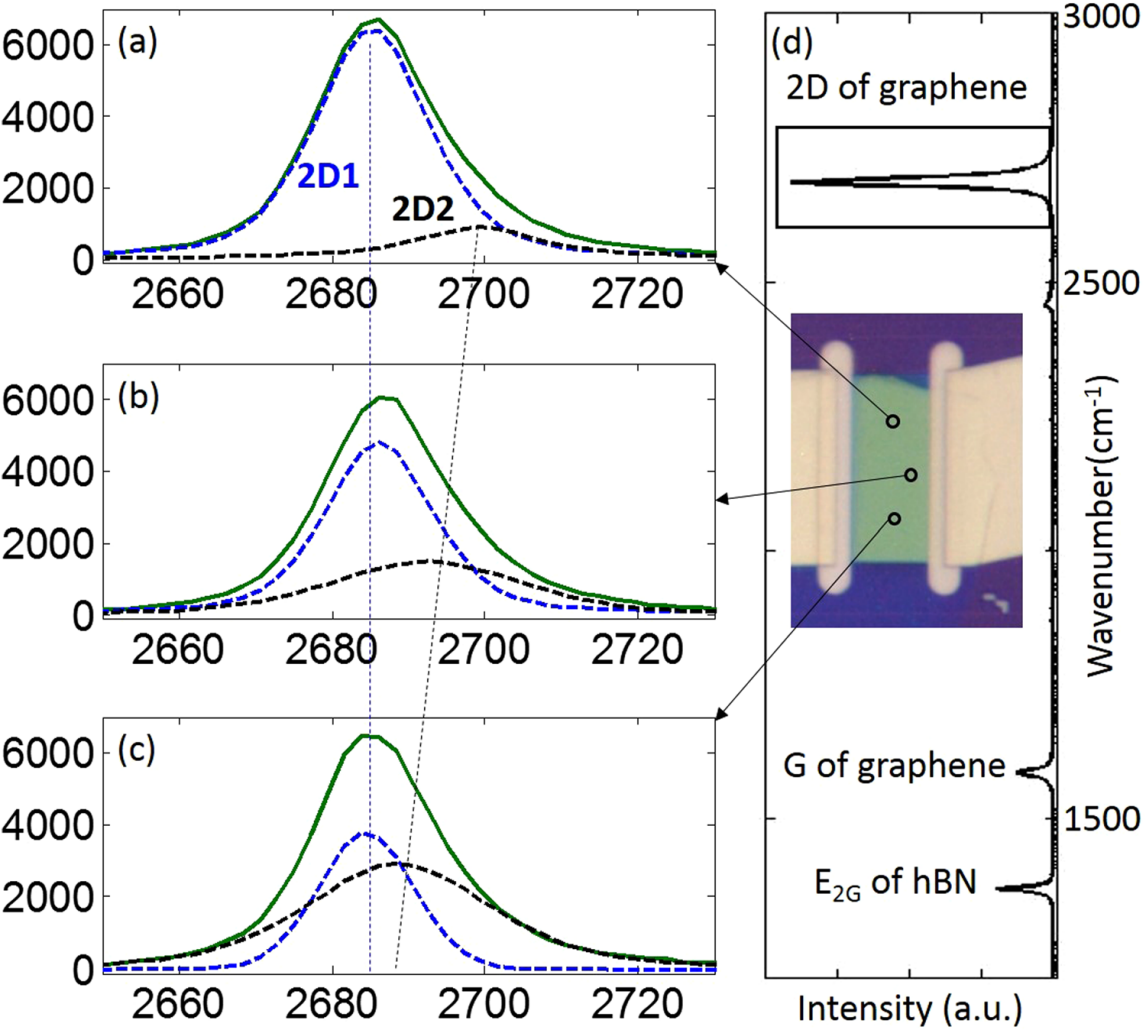
Examples of the variation of the 2D band at different spatial locations. (**a**–**c**) illustrates from top to bottom decreasing *2D*_1_ and 2*D*_*2*_ peak separation, change in relative intensity and varying peak energies. (**d**) shows a full spectrum with an inset of an optical image of the sample.

Raman data is collected from a graphene sample encapsulated in hBN. Examples of 2D spectra (λ = 532 nm) are shown in Fig. 2. The 2D peak is asymmetric and is fitted with two Voigt peaks where the lower energy peak is denoted *2D*_1_ (blue), and the higher energy peak is denoted 2*D*_*2*_ (black). The 2D peaks measured at different locations have different *2D*_1_ and 2*D*_*2*_ peak separations, linewidths, energies and intensities as shown in Fig. 2(a–c). We find similar variability in the 2D peaks across the sample. A full spectrum is shown in Fig. 2(d) with an insert of an optical image of the sample.

**Figure 3:**
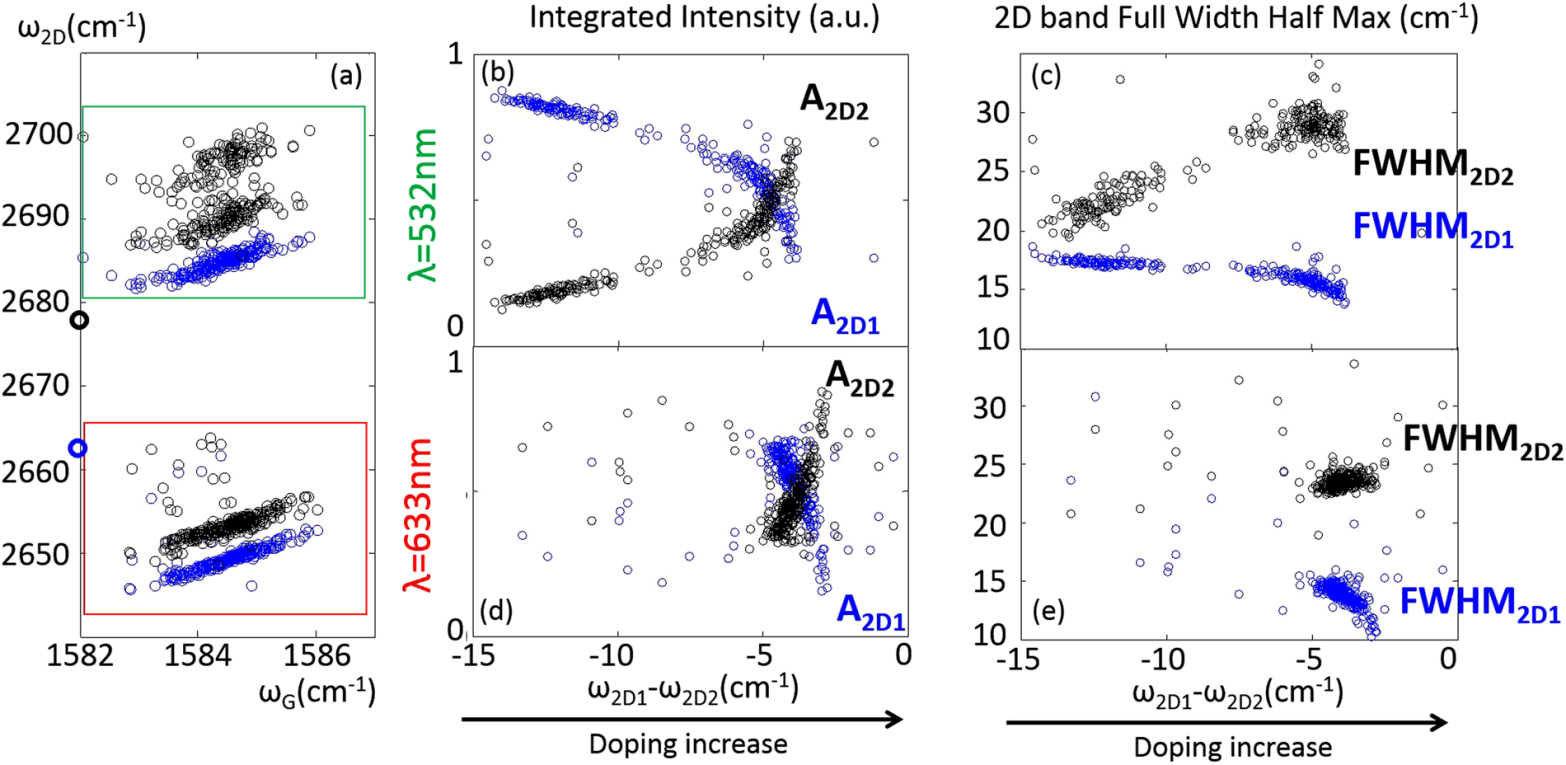
2D peak behaviour. (**a**) *2D*_1_-G correlation (blue) and 2*D*_*2*_-G correlation (black) for λ = 532 nm and λ = 633 nm for the hBN encapsulated graphene. For λ = 532 nm, the *2D*_1_-G slope is 1.72, the 2*D*_*2*_ is clustered with two different linear correlations with slope of 2.2 ± 0.3 (*2D*_*2−*_) and 3.0 ± 0.4 (*2D*_*2+*_)). For λ = 633 nm *2D*_1_-G has slope of 2.3 and 2*D*_*2*_-G has a slope of 2.2. For comparison with free-standing graphene we have included in bold symbols values for λ = 532 nm extrapolated from reference 18. (**b**–**e**) Scaled *2D*_1_ (blue) and 2*D*_*2*_ (black) normalized integrated intensities (A_2D2_ and A_2D1_) and their linewidths (FWHM_2D2_ and FWHM_2D1_) plotted against *2D*_1_ and 2*D*_*2*_ peak separation.

In order to evaluate the range of 2D spectra across the sample, we plot all the data measured on the same sample for two laser lines, λ = 532 nm and λ = 633 nm, in a 2D versus G diagram which reveals the contribution to the peak position due to strain or charge density^25^. Figure 3(a) shows *2D*_1_ (blue symbols) and 2*D*_*2*_ (black symbols) versus the corresponding G peak position where the different data sets are boxed with their respective laser colour. The red laser selects shorter *q* vectors through the DR mechanism and therefore has a significantly lower 2D energy than the green laser, as expected. Both data sets exhibit strain lines, indicating some compression^25,26^. We first consider Fig. 3(a) for λ = 532 nm. The lower energy *2D*_1_ peak has a well-defined strain-induced slope of 1.7 ± 0.13, very close to the calculated value for uniaxial strain/compression in a random lattice direction^25,26^. The strain variation across the sample is on the order of ±0.1% which is small enough not to cause strain-induced peak-splitting^27^. The presence of the small strain variation will increase the linewidth only marginally^26^. The higher energy peak 2*D*_2_ does not fall along a single, well defined line. Instead, the data falls mainly in two groups: a lower 2*D*_*2−*_ vs G, and a higher *2D*_*2*+_ vs G_._ Despite there being two *2D*_*2*_ groups, there is only one *2D*_1_ line_._ (Supplementary Information (SI) Fig. S1). The slopes measured for *2D*_*2*−_ vs G and *2D*_*2*+_ vs G are 2.2 ± 0.3 and 3.0 ± 0.4 respectively. The variation and range of the strain slopes matches earlier studies of graphene/hBN sandwiches^28^. For reference, 2D1 and 2D2 values for λ = 532 nm for freely suspended graphene has been included in Fig. 3(a) ^18^.

Here we are not interested in the strain *per se*, but rather the effect of low charge doping on the graphene Raman peaks. The charge present is much lower than can be evaluated with help of a 2D versus G plot^25^, or by the G band linewidth or peak position^23,24^ (SI Fig. S2 (a)). Instead of attempting to remove the strain component from the 2D peak behaviour (requiring exact knowledge of the strain slope and ${\omega }_{2{D}_{0}}$ position) we analyse the 2D characteristics versus the 2D peak splitting, since the split is a direct measure of doping and vanishes as doping increases^18^. Figure 3 (b–e) shows the normalized intensity and line width evolution as a function of 2D peak splitting for λ = 532 nm, top, and λ = 633 nm, bottom panels. Here we have chosen the *x*-axis to increase with increasing doping, as ${\omega }_{2{D}_{1}}-{\omega }_{2{D}_{2}}$, with large 2D peak split to the left defined as “pristine”, here meaning close to the CNP^18^, and higher charge density (small peak split) to the right. Figure 3(b) and (d) show the relative integrated intensity of *2D*_1_ and 2*D*_*2*_ band versus peak splitting, and Fig. 3(c) and (e) show how the linewidths of *2D*_1_ and 2*D*_*2*_ evolve as the splitting decreases. Figure 3(b–e) demonstrate that the normalized integrated intensity and the linewidth for each peak are strongly correlated with the peak splitting. The behaviours are qualitatively similar for both laser lines. The largest difference is that the 2D peak split range is much larger for λ = 532 nm, and the similar behaviour for λ = 633 nm is reproducibly compressed to a much smaller peak split variation for the identical sample scan area.

We establish a quantitative relationship between 2D peak splitting and doping by assuming a linear relationship between split and charge, and comparing means and standard deviations between the two. Transport measurements on a similarly prepared sample show that the mean doping level is 1.4 × 10^11^ cm^−2^ based on the back-gate voltage at the CNP. The charge variation is 0.4 × 10^11^ cm^−2^ given the residual conductivity at the CNP (see supporting information section 2). Comparing with our Raman data, which has a mean and standard deviation of −8.1 and 3.6 cm^−1^ for the 2D peak splitting, we find that for each cm^−1^ of 2D peak splitting, the doping changes by 0.11 × 10^11^ cm^−2^ making the 2D peak splitting a very sensitive probe of local charge density. Extrapolating linearly, we also find from this relationship that the CNP corresponds to a 2D peak splitting of 20.4 cm^−1^ (see supporting information section 3). While we know the mean and standard deviations for the doping in the sample used for transport measurements with high precision, and we know the mean and standard deviations for the 2D peak splitting for the sample used for Raman measurements, there is sample-to-sample variation which introduces uncertainty in our values for the slope of the linear correspondence and 2D peak splitting at CNP. We estimate a roughly 5% sample-to-sample variation, which translates into uncertainty in charge per wavenumber of 0.11 × 10^11^ ± 0.02 × 10^11^ cm^−2^ per cm^−1^, and 20.4 ± 1.1 cm^−1^ for the 2D peak splitting at the CNP.

While it would be advantageous to use electrostatic field gating to confirm, attempts to control the charge level via back-gating samples for optical measurements are complicated due to light-induced charge screening of the gate voltage for graphene on hBN. This is because light illumination on a gated graphene/hBN sample on SiO_2_/Si produces optical excitations of defect transitions in hBN and charge transfer to the graphene. In fact, light exposure during gating quickly returns the graphene to the charge neutrality point, undoing the gate doping^29^. The sign of the charge is also undetermined, but recent measurements^30^ on ultraclean samples have shown a symmetric electron and hole blue-shift of the 2D peak (single peak fitting) for charge density up to 2 × 10^12^ cm^−2^. Hence, the 2D peak response for holes and electron doping is indistinguishable on the Raman spectra at such low charge density. Early gated Raman measurements^31^ on graphene on SiO_2_ showed a weak difference between electron vs hole doping below 10^12^ cm^−2^ in contrast to the newer easurements^30^. This illustrates the need for excellent charge homogeneity to observe the low charge density effects achieved on suspended or hBN encapsulated graphene.

### Intensity, and the inner and outer processes

Generally, a high density of vector nesting creates a stronger Raman peak. This would indicate that the outer process, with flatter parts of the trigonal warping of the iso-energy contour matched by the phonon contour, has a stronger contribution to the Raman spectra than the inner process^15^, see Fig. 1(c). However, calculations including quantum interference and the *q*-dependent scattering matrix elements of the phonon intensity have shown that the inner process dominates despite the stronger vector nesting for the outer process^11–13^. Our results show the *2D*_1_ peak with the strongest intensity at the lowest doping level. A higher *2D*_1_ intensity has also been observed experimentally previously on both suspended^15,16,18^ and hBN encapsulated^17^ graphene. Based on the observed and predicted higher intensity for the inner process for pristine graphene, we tentatively assign the *2D*_1_ peak as originating from the inner process, and show below how this assignment is supported by the results. Figure 1(c) illustrates this assignment with the inner phonon having the lowest phonon energy. From previous work we know that the inner phonon vector *q*_*i*_ is larger than the outer vector *q*_*o*_, but the inner *KΓ* phonon dispersion is flatter than the steeper outer *KM* phonon branch^10,11,32^, as illustrated schematically in Fig. 1(c). In the schematic of Fig. 1(c), the outer phonon branch has a high enough slope to result in a higher phonon energy, consistent with the assignment of *2D*_1_ as the inner process.

Intensity behaviour as a function of charge has not yet been explored theoretically. We suggest that the observed switch in intensity from *2D*_1_ to 2*D*_*2*_ with increased charge screening may be an effect of changing wave vectors and phonon energies that will affect both the scattering matrix terms in the numerator, as well as the denominator of the Raman intensity scattering expression^12^, which will change the relative strengths of the *2D*_1_ and 2*D*_*2*_ peak due to quantum interference. Regardless of the cause for the intensity behaviour of the two 2D peaks, it is clear that added charge to graphene in hBN changes the dominant intensity from the *2D*_1_ peak (inner) to the 2*D*_*2*_ peak (outer) with increasing charge.

### Linear dispersion model

The key to the split of the 2D peak into *2D*_1_ and 2*D*_*2*_ components and their linewidths is the ratio of the phonon velocity *v*_*ph*_ to the Fermi velocity *v*_*F*_ for the inner and outer process. Using the DR mechanism and the simplifying assumption of perfectly linear electronic and phonon dispersions around the *K* and *K’* points (but with different slopes for the inner and outer directions)^10–13^, and using *v*_*ph*_ ≪ *v*_*F*_, we have

1 $$q=\frac{{E}_{L}}{2\hslash {v}_{F}}+\frac{{E}_{L}-2(hc{\omega }_{{D}_{0}}+\hslash {v}_{ph}q)}{2\hslash {v}_{F}}\Rightarrow q=\frac{{E}_{L}-hc{\omega }_{{D}_{0}}}{\hslash {v}_{F}}{(1+\frac{{v}_{ph}}{{v}_{F}})}^{-1}$$

2 $$\frac{1}{2}{\omega }_{2D}={\omega }_{{D}_{0}}+\frac{\hslash {v}_{ph}q}{hc}={\omega }_{{D}_{0}}+\frac{{v}_{ph}}{{v}_{F}}\cdot (\frac{{E}_{L}}{hc}-{\omega }_{{D}_{0}})\cdot {(1+\frac{{v}_{ph}}{{v}_{F}})}^{-1}\approx {\omega }_{{D}_{0}}+\frac{{v}_{ph}}{{v}_{F}}\cdot (\frac{{E}_{L}}{hc}-{\omega }_{{D}_{0}})$$

3 $$hc\frac{\partial {\omega }_{2D}}{\partial {E}_{L}}=2\frac{{v}_{ph}}{{v}_{F}}{(1+\frac{{v}_{ph}}{{v}_{F}})}^{-1}\approx 2\frac{{v}_{ph}}{{v}_{F}}$$

4 $$\frac{{\omega }_{2D,o}-{\omega }_{2D,i}}{2}=(\frac{{v}_{ph,o}}{{v}_{F,o}}-\frac{{v}_{ph,i}}{{v}_{F,i}})\cdot (\frac{{E}_{L}}{hc}-{\omega }_{{D}_{0}})\cdot {(1+\frac{{v}_{ph}}{{v}_{F}})}^{-1}\approx (\frac{{v}_{ph,o}}{{v}_{F,o}}-\frac{{v}_{ph,i}}{{v}_{F,i}})\cdot (\frac{{E}_{L}}{hc}-{\omega }_{{D}_{0}})$$

5 $$\delta {\omega }_{2D,io}=4h\frac{{v}_{ph}}{hc}\delta {q}_{i,o}=4{(\frac{{v}_{ph}}{{v}_{F}})}_{i,o}\frac{{\gamma }_{i,o}}{hc}{(1+\frac{{v}_{ph}}{{v}_{F}})}^{-1}\approx 4{(\frac{{v}_{ph}}{{v}_{F}})}_{i,o}\frac{{\gamma }_{i,o}}{hc}$$

where ${\omega }_{{D}_{0}}$ is the D phonon energy (cm^−1^) at the *K* point (*q* = 0), *γ* is the imaginary part of the electronic self energy (eV), *h* is Planck’s constant, and *c* is the speed of light. The subscripts *i* and *o* refer to the inner and outer phonons, respectively. Equations 2–5 illustrate that the phonon energies, Eqn. (2); the dispersion with laser energy, Eqn. (3); the peak split, Eqn. (4); and the full width at half maximum (FWHM) linewidths, Eqn. (5), all depend on the ratio of the phonon to electron velocity in the inner and outer scattering processes. We use the results from the two laser lines to find the shift with laser energy^33^, *∂ω/∂E*_*L*_, of the *2D*_1_ and 2*D*_2_ band for “pristine” and charged graphene and use Eqn. (3) to determine the velocity ratio. For 2*D*_*2*+,_ the low intensity peak at CNP, the slope is 120 cm^−1^/eV, while the other ratios fall in the range of the values reported for graphene and carbon nanotubes ~88–110 cm^−1^/eV^22,33,34^. Using these ratios, we can also calculate ${\omega }_{{D}_{0}}$, which in turn can be used to determine how the phonon velocity changes with screening along the *KΓ* and *KM* directions. The results are tabulated in Table 1. The linear approximation gives the “local” slope between the phonon wave-vectors selected by the green and red laser. Hence, the different values of ${\omega }_{{D}_{0}}$ are a reflection of the *local* slopes. The phonon dispersion near *K* has a negative second derivative, so the actual ${\omega }_{{D}_{0}}$, which is the same for both the inner and outer process, will be below or equal to the smallest tabulated value, 1214 cm^−1^.

**Table 1:** Extracted values for the lower and higher 2D energy peaks.

Correlations				
variables	2*D*_1(+)_	2*D*_1(−)_	2*D*_2(+)_	2*D*_2(−)_
$\frac{\delta {\omega }_{2D}}{\delta {E}_{L}}(c{m}^{-1}/eV)$	97	97	120	103
$\frac{{v}_{ph}}{{v}_{F}}({10}^{-3})$	6.5	6.0	6.8	6.1
*ωD*_0_(*cm* ^−1^)	1233	1233	1214	1230
$\frac{1}{2}{\omega }_{2D}(c{m}^{-1})$	1343	1342	1349	1345
Δ*ω*(*cm* ^−1^)	110	110	135	115
$\frac{{\rm{\Delta }}\omega }{q}\propto {v}_{ph}$	$\frac{110}{{q}_{1}}$	$\frac{110}{{q}_{1}+{\rm{\Delta }}{q}_{1}}$	$\frac{135}{{q}_{2}}$	$\frac{115}{{q}_{2}+{\rm{\Delta }}{q}_{2}}$

The ± signs denote the “pristine” and charged Raman response, where “pristine” results are denoted with (+). *Δω* denotes the difference of the measured *½ω*_*2D*_ and the extracted value of ${\omega }_{{D}_{0}}$ at the *K* point (*q* = 0). Note that *2D*_1_ has overlapping values for *2D*_1_ pristine and charged graphene, but that the increased charge causes an increase in the *q* vector, *q*_1_ + *Δq*_1_, which indicate a lower phonon velocity.

**Figure 4:**
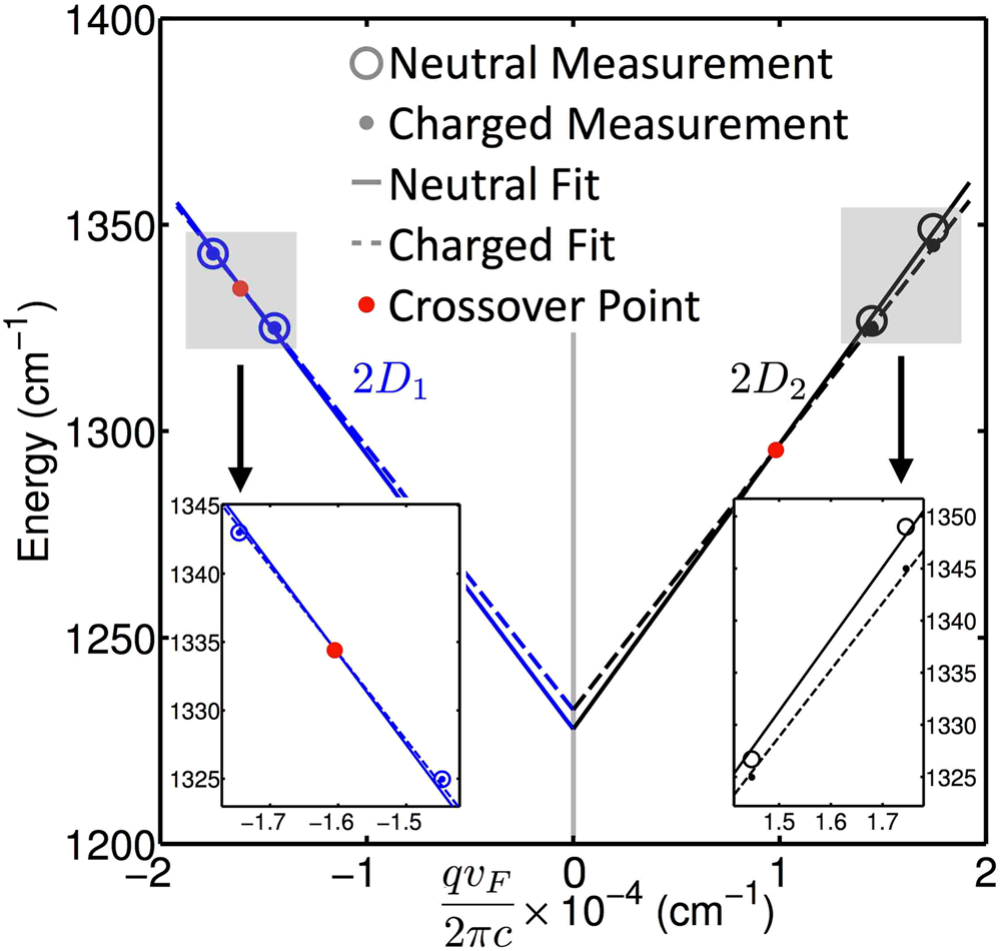
Scaled phonon dispersion. The figures shows the experimental data from Table 1 and the best fit linear model forcing a common for *2D*_1_ and 2*D*_*2*_ phonons. The phonon momentum on the *x*-axis is scaled so that all scaled *q* values are given directly by measurements. Note that the Fermi velocity scaling *q* is that of the particular case, i.e., inner or outer process, and uncharged or charged.

We can also fit the data with a linear model, detailed in SI, section 2, that forces a common ${\omega }_{{D}_{0}}$ as shown in Fig. 4. Here we are using a scaled *x*-axis, where instead of *q*, we are plotting the phonon dispersion versus *x* = *q*_*i*_ *v*_*F*,*i*_/2*πc*, which is directly measurable without knowing *v*_*F*_. This gives the exact scaled *q* values for *i* = 1 ~ 4, *i* representing red and green laser for uncharged and charged graphene, respectively. Note that by this construction, the laser energy sets the scaled *q* value *x*, see SI. The best fit values for ${\omega }_{{D}_{0}}$ are 1228 and 1233 cm^−1^ for uncharged and charged graphene, respectively, tabulated in SI Table S3.

Both linear models (local slope versus common ${\omega }_{{D}_{0}}$) exhibit the crossover in dispersion attributed to screening of the phonon dispersion, illustrated both in Fig. 1(c), bottom, and Fig. 4. The change in the local phonon dispersion with increased screening has been predicted by comparisons between the phonon dispersion using the GW approach for screened interactions, and density-functional theory that represents unscreened interactions^10,11^. The increase in ${\omega }_{{D}_{0}}$ and decrease in phonon velocities with increased charge is a direct indication of lifting of the *K*-point Kohn anomaly due to screening^10,11^ and a weakening of EPC^3^.

### Split peak phonon energy versus charge density

It is counterintuitive that the *2D*_*2*_ phonons *decrease* with increased charge (Fig. 3a), since increased screening lowers the Fermi velocity and increases the DR-selected *q* vector from *q* to *q* + *Δq*, which typically corresponds to a higher *ω*_*D*_^17,19,20^. The linear models above demonstrate that the decrease in *2D*_*2*_ with increased charge is due to a crossover of the unscreened and screened phonon dispersion, as seen in calculations^10,11^. The dispersion crossover under different charge screening conditions enables the decrease in phonon energy on the outer branch, while the slope and crossover of the inner branch conspires to yield a basically unchanged *2D*_1_ phonon energy as indicated by the two *q* values, *q*_*i*_ and *q*_*i*_ + *Δq*_*i*_ (Fig. 1c). The reduction of the phonon velocities is commensurate with lifting the Kohn anomaly at *K* with charge screening. The significant decrease in peak splitting for λ = 633 nm compared to λ = 532 nm from 15 to 4 cm^−1^ for graphene near the CNP is indicative of a sub-linear dispersion (negative second derivative) of the inner branch, as expected^10,11^.

### Linewidths

As indicated in Eqn. (5), the linewidths of *2D*_1_ and 2*D*_*2*_ are not indicative of the phonon lifetime, but rather determined by the electronic lifetime (imaginary part of the self-energy) and ratio of the phonon to electron velocity. However equation 2 provides a convenient mapping between scattered photon energy and phonon energy, and from this perspective the linewidth is pictured as a blurring of the DR phonon contour to a width, *δq*_*i*,*o*_. Phonons that fall within the blurred contour make a significant contribution to the 2D peak, and we will regard them as participatory phonons, and the blurred line as the participatory contour. The middle of the participatory contour is mapped almost perfectly to the triangularly warped iso-energy phonon contour with the “corners” pointing towards *Γ* rather than *M*. The width of the participatory contour in the *KΓ* direction (inner), *δq*_*i*_, has been calculated^12^ to be broader than the width in *KM* direction (outer), *δq*_*o*_, which provides a method for assigning 2D peaks to inner and outer processes. Using equations (1) and (5) and our measurements we relate the ratio of inner to outer participatory contour widths to the ratio of inner to outer linewidths, outer to inner phonon to Fermi velocity ratios, and outer to inner electron velocities $\frac{\delta {q}_{i}}{\delta {q}_{o}}=\frac{\delta {\omega }_{i}}{\delta {\omega }_{o}}\frac{{({v}_{ph}/{v}_{F})}_{o}}{{({v}_{ph}/{v}_{F})}_{i}}\frac{{v}_{F,o}}{{v}_{F,i}}$. To make a proper comparison with theory computed assuming zero doping, we use our data in Table 1 and electron transport data to estimate the ratio of contour widths at the CNP (see supporting information section 3 for details). Assuming the 2D_1_ peak corresponds with the inner process and the 2D_2_ peak corresponds with the outer process we find this ratio to be 1.3, but if the correspondence between peaks and inner/outer is reversed, this ratio is only 0.8. Since theory predicts the ratio of contour widths to be greater than one^12^, our data supports the assignment of *2D*_1_ as originating from the inner process.

Charge affects the 2D linewidth through two primary mechanisms: scattering and phonon velocity renormalization. Eqn. (5) shows that the linewidth is proportional to the imaginary part of the electronic self-energy and the phonon velocity. Experimentally we can only measure the phonon to electron velocity ratio, which we will use as a proxy for the phonon velocity keeping in mind that the electron velocity slightly decreases with charge^9^. For λ = 532 nm, the “pristine” linewidths of the *2D*_1_ and 2*D*_2_ peaks are almost equal, but as charge increases the *2D*_*2*_ linewidth steadily rises while the *2D*_1_ linewidth remains level until the peak split nearly reaches its minimum, at which point the linewidth surprisingly decreases (Fig. 3(c)). It is generally expected that the linewidth should increase with charge because the electronic self-energy increases^12^, which makes the nearly constant 2D_1_ linewidth surprising. However, taking into account the change to the phonon velocity we’ll see that our observations are consistent with increased electronic self-energy. Our data shows that the phonon to electron velocity ratio for the *2D*_1_ peak decreases with charge (Table 1), which, given that charge reduces the electron velocity, implies that the phonon velocity must decrease as well. Our observation of a nearly flat 2D_1_ linewidth versus charge is then consistent with a self-energy that increases with charge, but compensated for by a reduced phonon velocity. For the 2*D*_*2*_ peak, the phonon to electron velocity ratio also decreases with charge, but in this case the linewidth increases. We offer two explanations for this difference: 1) The electronic self-energy is momentum dependent, and the momenta that contribute to the *2D*_1_ and 2*D*_2_ peaks are different. Our observations are then consistent with an electronic self-energy that increases more greatly with charge for momenta that contribute to the *2D*_*2*_ peak than momenta that contribute to the *2D*_1_ peak. 2) The Fermi velocity decreases with charge less dramatically along the direction of the 2*D*_2_ process than the 2D_1_ process. If this is the case, then the *2D*_*2*_ phonon velocity reduces by smaller amount than the *2D*_1_ phonon velocity, and will result in a 2*D*_*2*_ linewidth that increases more rapidly with charge.

## Discussion

The results presented here on hBN encapsulated graphene have both similarities and differences to suspended graphene. Both types of samples exhibit a decrease in the 2D double peak split with increased charge, and the lower energy peak, *2D*_1_, has the highest intensity. The screening of the *K*-point Kohn anomaly with increasing charge has not yet been addressed experimentally on suspended graphene. A significant difference is the substantial decrease in the peak split at the CNP for λ = 633 nm, not observed for suspended graphene where the 2D peak-split stays nearly constant (~13 ± 3 cm^−1^) over a laser energy range from 1.5–2.7 eV^15,18^. This indicates that the dielectrically screened graphene is less well fit with a linear model than suspended graphene: The peak split ${\omega }_{2D1}-{\omega }_{2{D}_{2}}$ between inner and outer phonon energies is proportional to the difference between the inner and outer velocity ratio, Eqn. (4), which is constant in a linear model. Based on the linewidth as a function of *E*_*L*_, Berciaud *et al*.^18^ dismissed the theory of the peak split originating from the difference in the inner and outer 2D phonons. The phonon linewidth is expected to decrease with lower laser energy, since *γ*_*eh*_ *∝* *E*_*L*_, and Berciaud *et al*.^18^ observed an increasing linewidth for laser line energies below ~1.7 eV for suspended graphene. We note that it is possible that the predicted increase in phonon dispersion slope closer to the *K* point^10,11^, probed with lower laser energies, dominates over the decrease in electronic linewidth, *γ*_*eh*_.

The lifting of the *K*-point Kohn anomaly with screening observed here is a sign of the weakened EPC^3^. Excitation of D band phonons by electrons is a fundamental bottleneck for ballistic transport at high fields^35^. Hence, it is possible that encapsulated graphene with low and uniform charge screening could have better transport properties at high fields than suspended graphene if the screening sufficiently reduces the strength of the EPC of the D phonons, without introducing other scattering mechanisms.

## Summary

In conclusion, we have used Raman spectroscopy of graphene encapsulated in hBN to explore the intrinsic double peaked 2D phonon behaviour under low charge screening. The analysis of the 2D data as a function of *2D*_1_ and 2*D*_*2*_ peak separation removes influence of strain, and reveals strong correlations between charge, peak intensities, peak positions and linewidths. The peak split can be used to estimate the charge density with sensitivity ~ 10^10^ cm^−2^ per cm^−1^ 2D peak split, an improvement of two orders of magnitude compared to use of the G band Kohn anomaly at the *Γ* point. Hence, the 2D peak split could be very informative for detecting low amounts of charge, with the caveat that the peak split with charge behaviour is known for the particular substrate screening and laser line used. The method also reveals information about the origin of the double peak and the effect of increased charge, for example the intensity shifts from the inner to the outer phonon with increased charge. We associate the lower energy *2D*_1_ peak with the so called inner process, and the higher energy 2*D*_*2*_ peak with the outer process. Even the low amount of charge puddling found in these samples is enough to significantly alter the strength of the D band Kohn anomaly at the *K* point.

## Methods

The encapsulated graphene sample was fabricated at Columbia University by Carlos Forsythe with their pick-and-place method that avoids direct contact between the pick-up polymer and graphene layer or the accompanying hBN surfaces that contact the graphene^21^. This particular sample did not have functioning edge contacts, but similar samples have a room temperature mobility of ~3.2 × 10^4^ cm^2^/Vs.

The collected 2D Raman spectral map is fitted with two Voigt peak functions at all different locations on the hBN encapsulated graphene. Different incident laser photon energies, green laser with wavelength of 532 nm (*E*_*L*_ = 2.33 eV) and red laser with wavelength of 633 nm (*E*_*L*_ = 1.96 eV), are applied at 1 µm by 1 µm spacing with a laser spot of approximately 1µm^2^ with typical spectra shown in Fig. 2. The incident laser power was limited to 1.2 mW to avoid heating.

### Data Availability

The datasets generated during and/or analysed during the current study are available from the corresponding author on reasonable request.

## Electronic supplementary material

Supplementary Info
