# Supplementary material for: 2D Raman band splitting in graphene: Charge screening and lifting of the K-point Kohn anomaly
Source: Sci Rep. 2017 Oct 19;7:13539. doi: 10.1038/s41598-017-13769-3 (PMC5648804; doi:10.1038/s41598-017-13769-3)
Supplement: Supplementary file 1 — Supplementary Info [file 41598_2017_13769_MOESM1_ESM.doc]

2D Raman band splitting in hBN encapsulated graphene: Charge screening and lifting of the *K*-point Kohn anomaly

Supporting Information

Xuanye Wang†, Jason W Christopher* and Anna K Swan†*

†Department of Electrical and Computer Engineering, Boston University, 8 St Mary’s St, Boston Massachusetts 02215, United States of America

*Department of Physics, Boston University, 590 Commonwealth Ave, Boston Massachusetts 02215, United States of America

**1. Raman Study**

1. ***2D_1(+)_* v.s. *2D_2+_* compared to *2D_1(-)_* v.s. *2D_2_-***

The distribution of the *2D_2_* band falls mainly into two data groups. The corresponding *2D_1_* data is also separated according to the *2D_2_*. The *2D_+_* (near CNP) and *2D-* (accidental charge doping) data are shown separately in Fig. S1 (b) and (c) respectively.


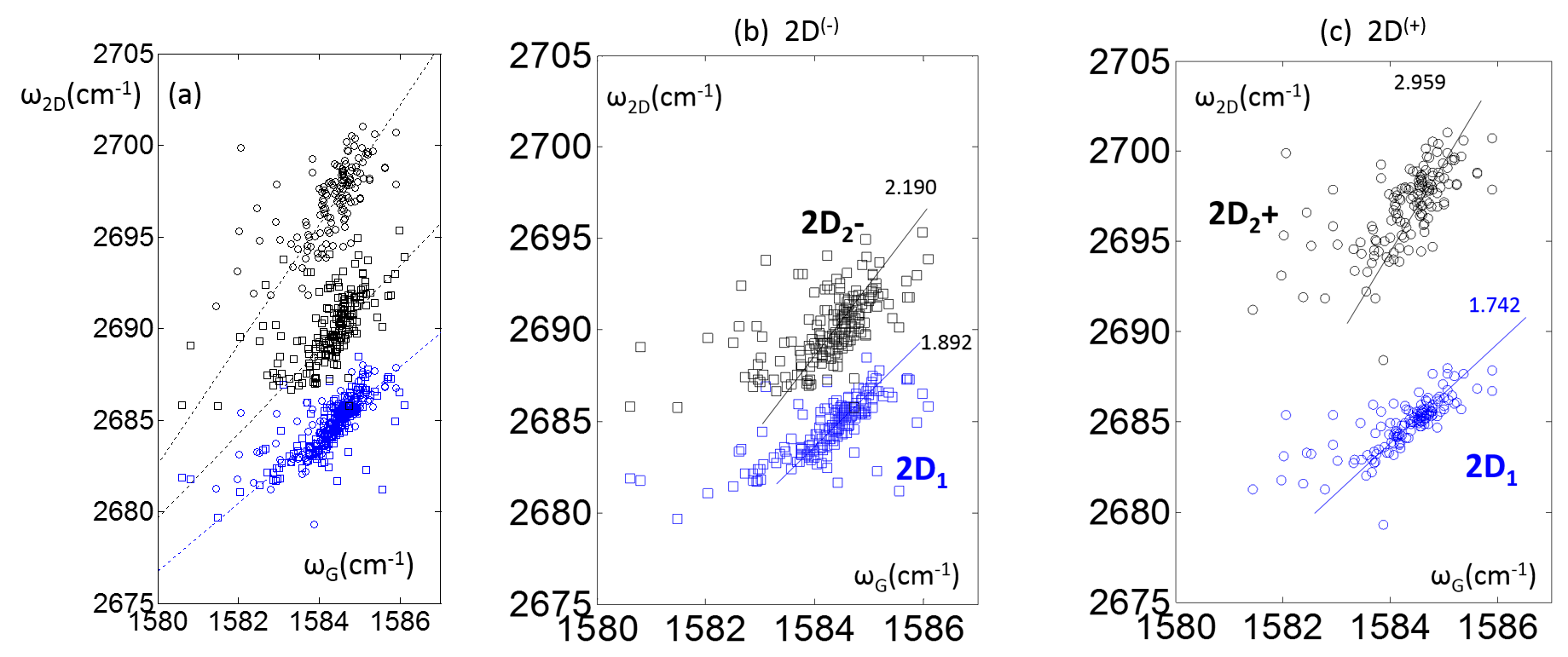


**Figure S1**. 2D vs G correlation of *2D_1_* and *2D_2_* separated according to peak split. Blue black data points correspond to *2D_1_* and *2D*_2_ bands respectively as in the main text Fig. S3. Square and circular data points correspond to 2D(-) (charged) and 2D_(+)_ (near CNP), respectively. (a) Full dataset. (b) Accidentally doped: *2D_2-_* and corresponding *2D_1(-)_*. (c) Near CNP: *2D_2+_* and corresponding *2D_1(+)_*. The slope of the *2D_2_* bands vary from 2.19 to 2.96, while the slope of *2D_1_* bands vary from 1.74 to 1.89.

1. **2D vs G, and linewidths**

Fig. S2 shows the distribution of the *2D-G* correlation for 532 nm wavelength laser excitation. The G band linewidth shows a tight distribution centred at 15 cm^-1^, which confirms that the overall charge density in the encapsulated graphene does not exceed 10^12^cm^-2^ [1].


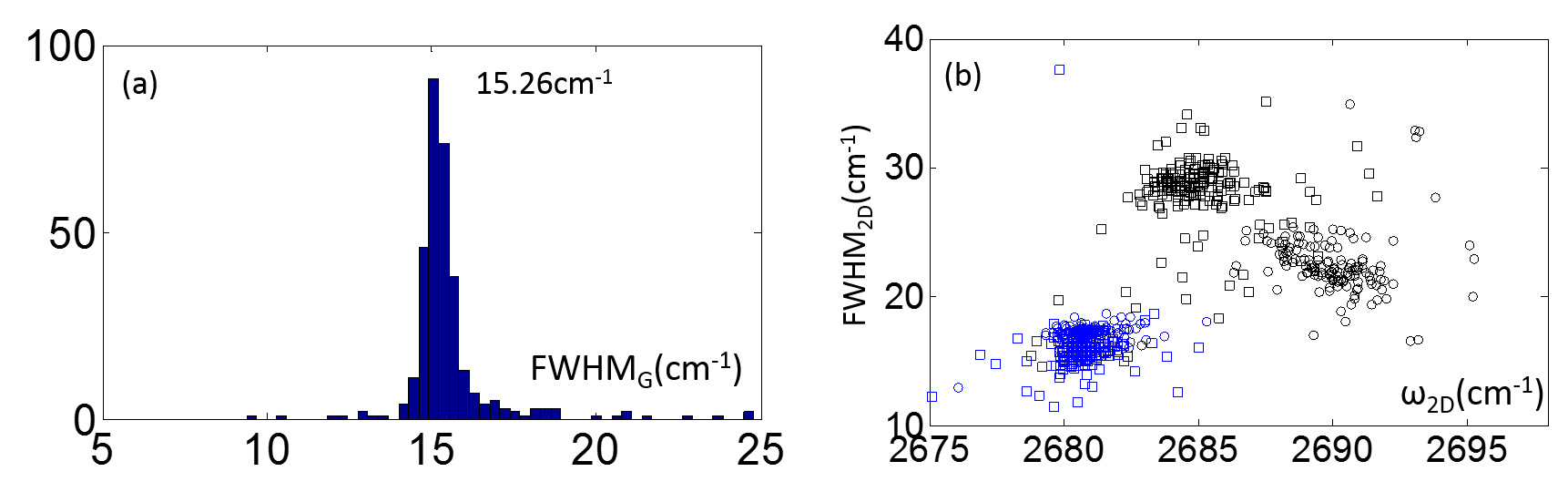


**Figure S2**. Raman G and 2D linewidth data for graphene encapsulated in hBN using λ = 532 nm laser excitation. (a) Histogram of G band linewidth. (b) 2D band linewidth versus 2D band position after removing strain induced peak shift. The legends are the same as used in Fig. S1.

1. **Non-scaled 2D band intensities and relative intensity ratio**

The overall 2D band integrated intensity remains relatively constant regardless of charge (Fig. S3). The integrated intensity ratio *A_2D1_ /A_2D2_* decreases linearly as a function of 2D split, shown in Fig. S4.


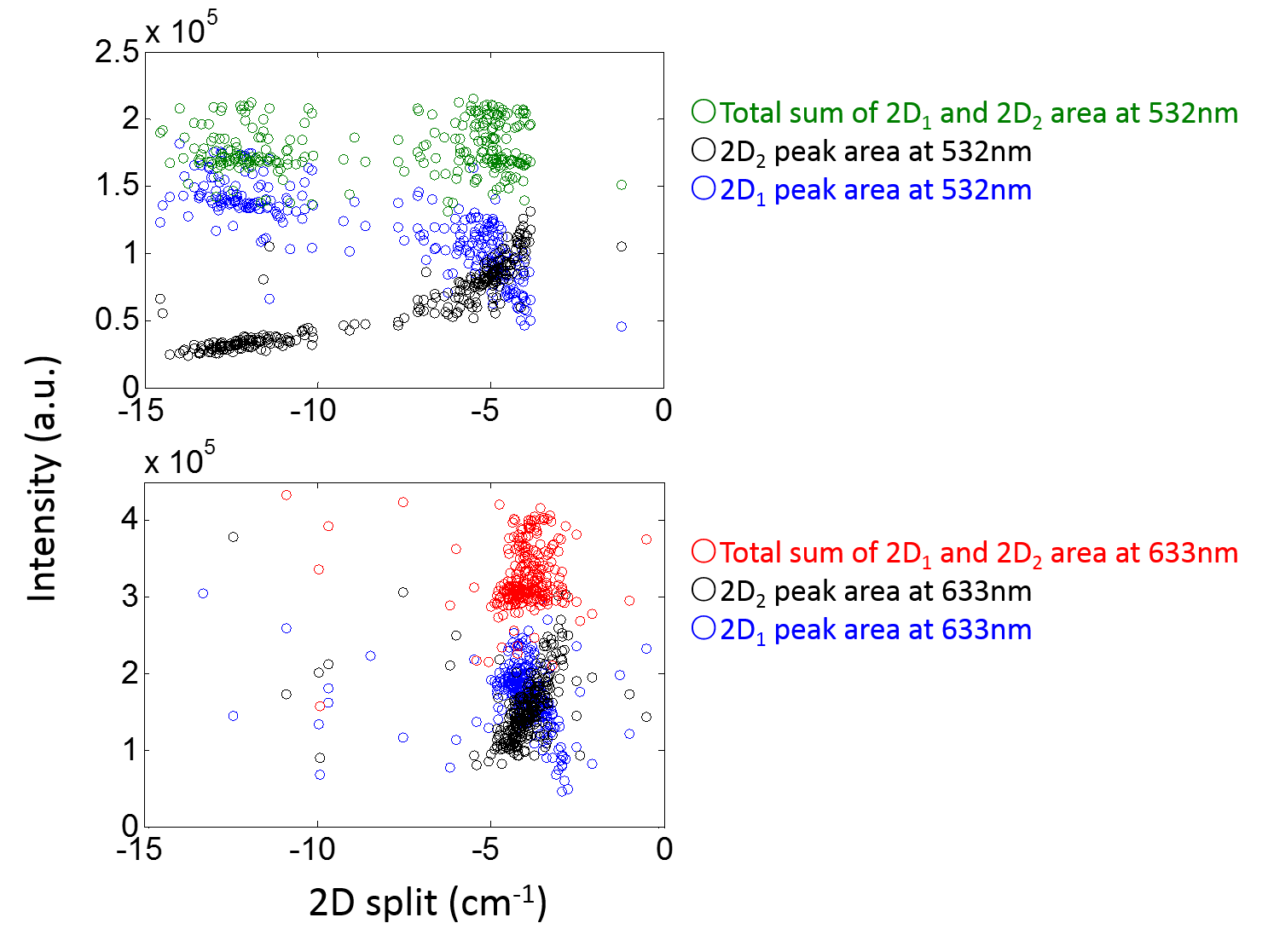


**Figure S3**. 2D band overall integrated intensity (the integrated intensity of *2D_1_* and *2D_2_*) versus 2D band splitting for (a) 532 nm, (b) 633 nm.


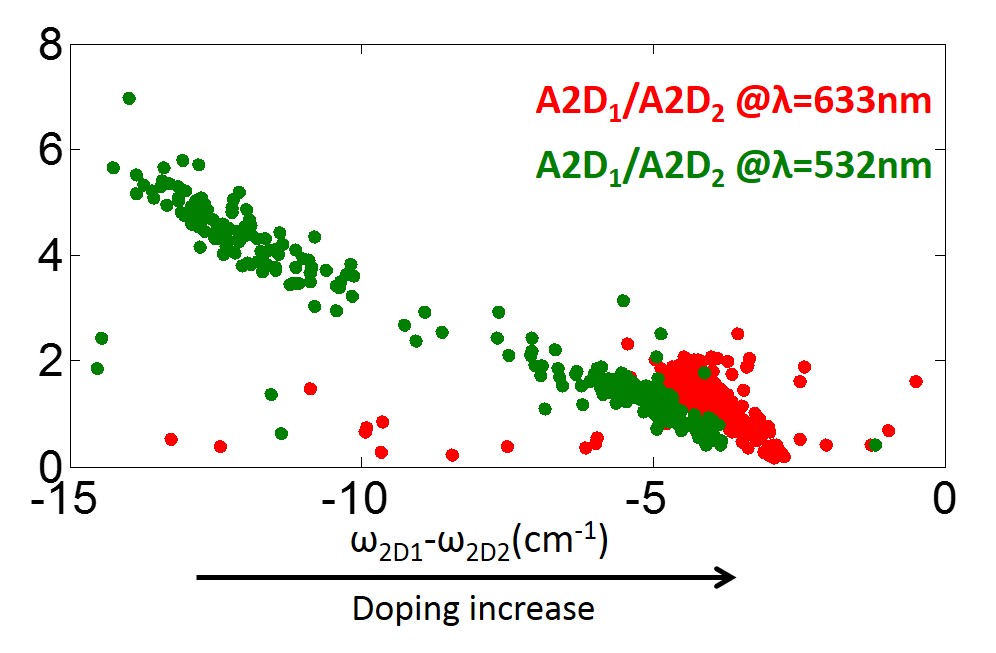


**Figure S4**. *2D_1_* and *2D_2_* integrated intensity ratio as a function of split under different incident photon energy. These ratios show linearly decreasing correlation as the split gets closer to zero for both excitation wavelengths.

1. **Linewidth fitted by two 2D sub-bands and only one peak**

The linewidths of 2D_1_ and 2D_2_ peaks are plotted on top of each other to compare their behaviour in Figure S5.


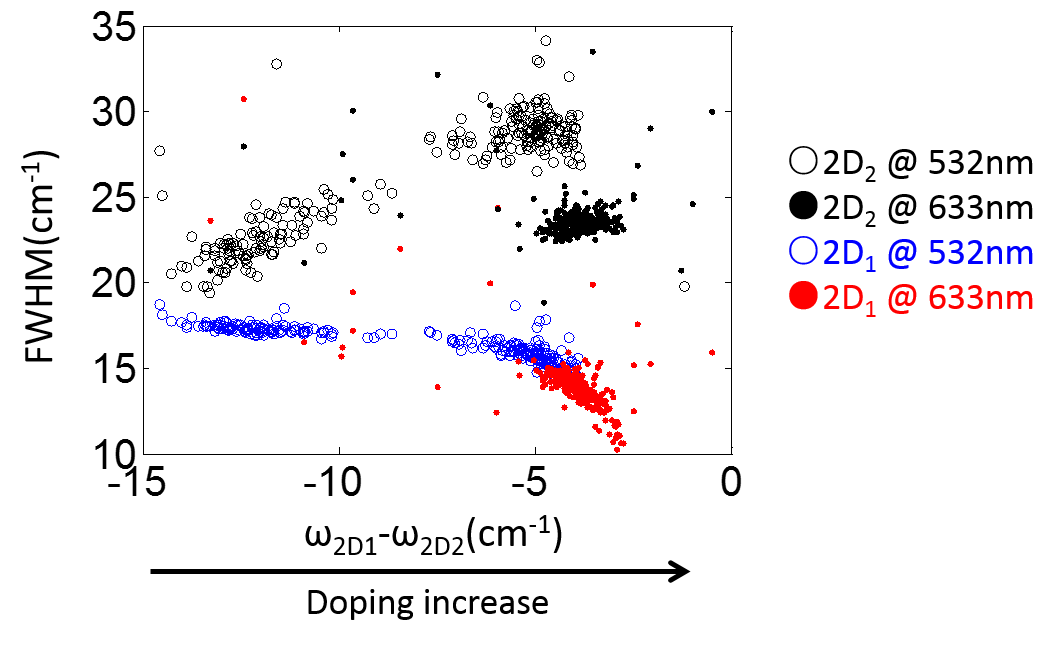


**Figure S5**. 2D band linewidth versus 2D band splitting under conditions that the spectra are fitted by one single peak and two split *2D_1,2_* peaks.

1. **Spatial map of 2D band split**

The spatial map of 2D band split is shown in Fig. S6. Even though we could see the low doping (large split, shown in yellow) and high doping (smaller split, shown in red) regions forming individual islands, no clear feature of doping domains can be concluded based on this 2D band split map. Here we use the data measured under 532nm laser excitation with relatively larger split to facilitate visualization.


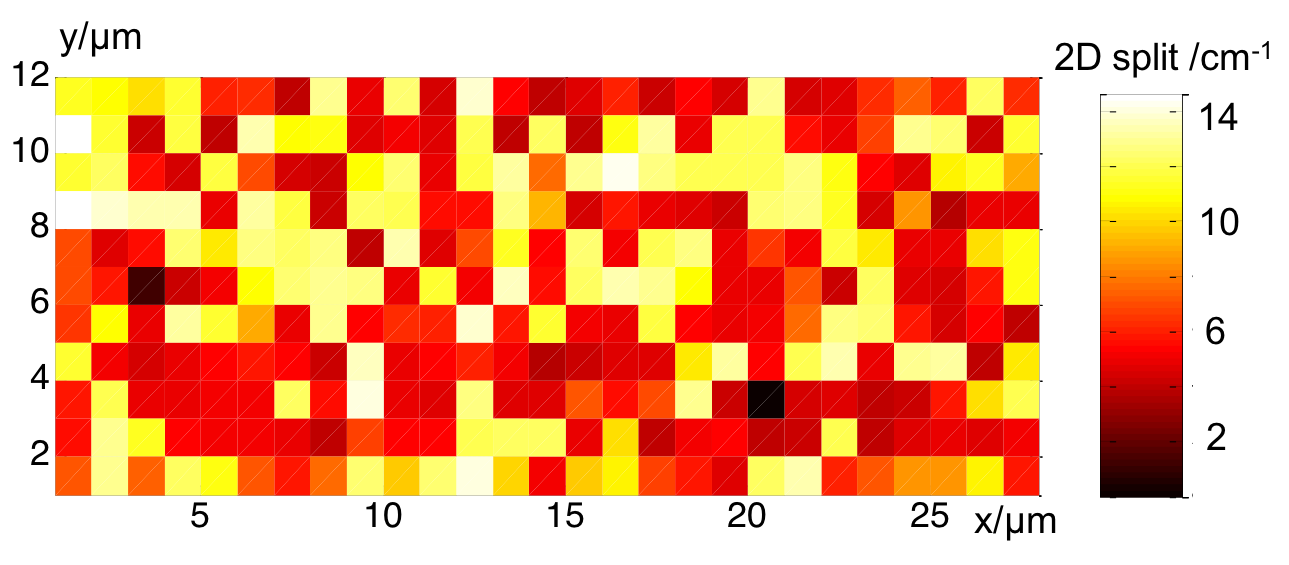


**Figure S6**. 2D band doping map under 532 nm laser excitation.

1. **Spectra Fitting – Single Peak and Double Peak**

We compared the residue of the 2D band spectra fitted by single 2D peak and double 2D peaks. Figure S7 shows the comparison of data acquired using 532nm laser line at two different locations with different split values (5.10cm^-1^ and 11.22cm^-1^). Both fits are done using Voigt function. In both cases, the residue of the data fitted by single 2D peak shows a clear structure comparing to the close to zero residue fitted using double 2D peaks.


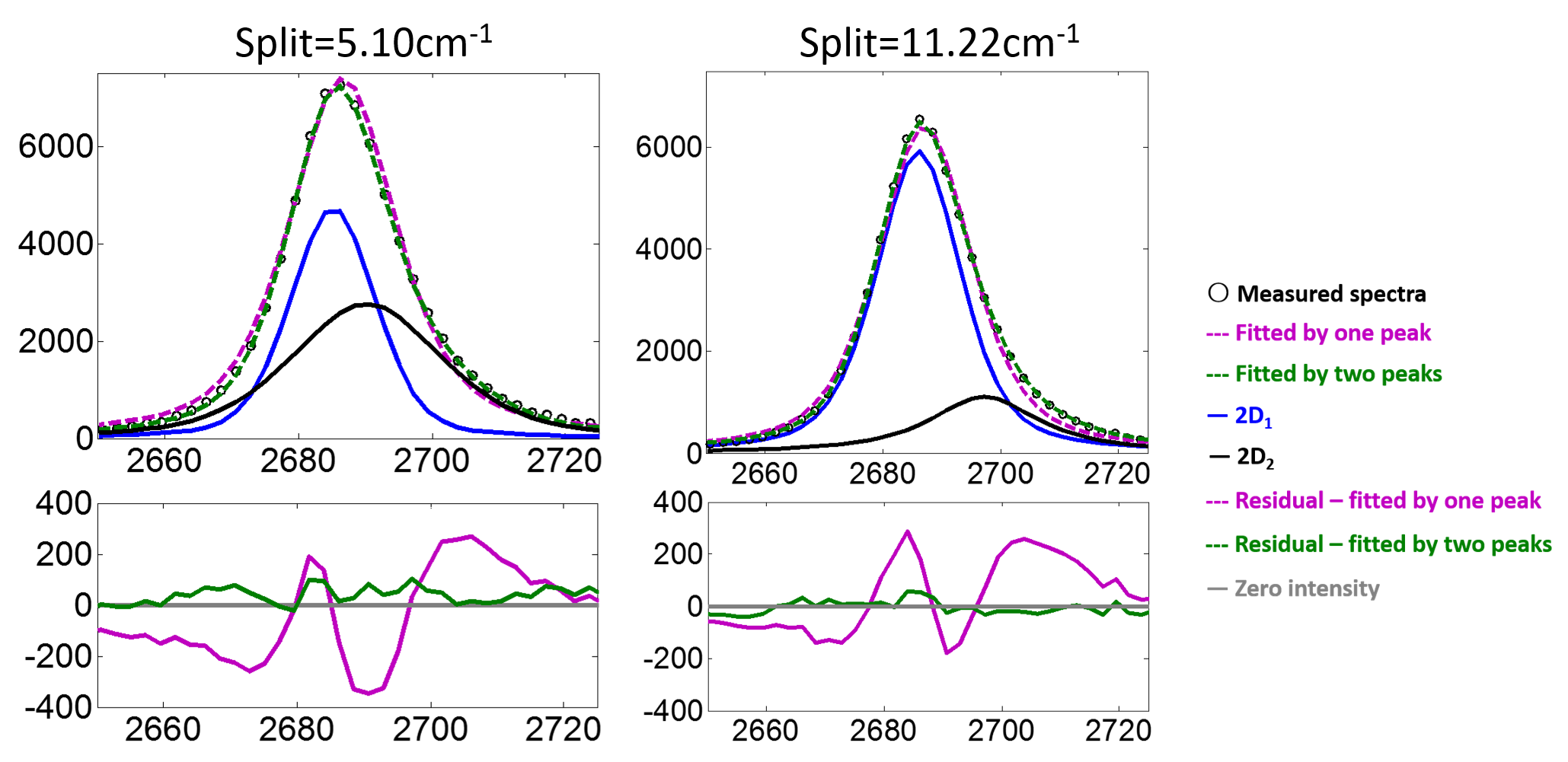


**Figure S7**. Residue of the spectra fitted by single 2D peak and double 2D peaks at different locations.

We also compare the 2D-G behaviour between the double 2D peaks fitted data discussed in the main text and data fitted using one 2D peak. The 2D-G correlation with 2D band fitted using one peak is plotted using green data points on top of the previously discussed 2D_1_ and 2D_2_ lines, shown in Fig. S8(a), along with green dashed line showing a linear fit of 2D-G correlation, shown in Fig. S8(b). The dashed green line has a slope (1.78±0.17) very close to previously report values^3,4^, with a relatively tight distribution along the fitted linear correlation less than 1cm^-1^, which is another indication of un-distinguishable low doping value using the doping vector method^3^.


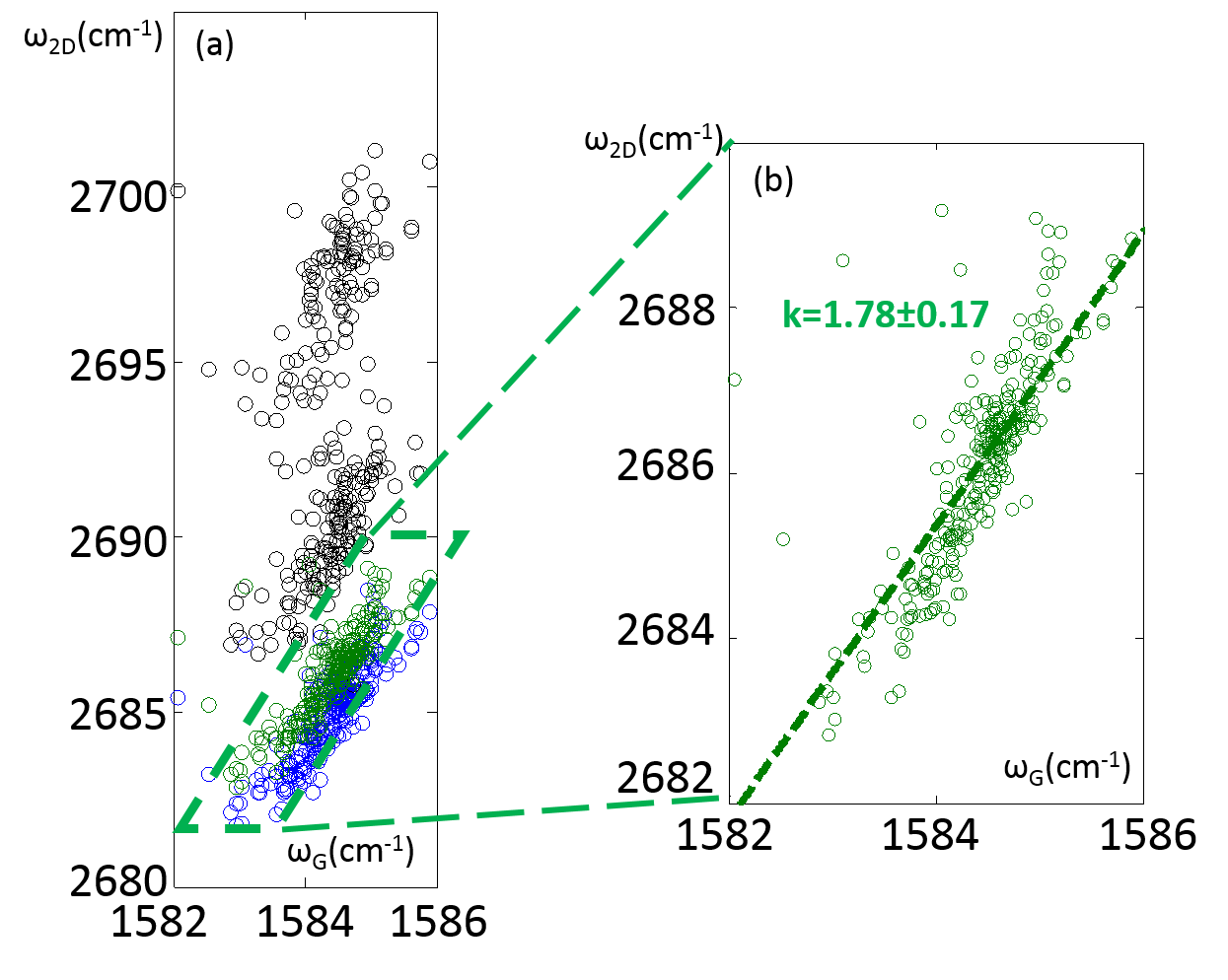


**Figure S8**. (a) Comparison of the 2D-G correlation with 2D band spectra fitted using double 2D peaks (with 2D_1_ and 2D_2_ using the same notation discussed in the main text) and fitted using single 2D peak (green data points). (b) 2D-G correlation with 2D band fitted using single 2D peak. The fitted linear correlation is displayed in dashed green line with slope of 1.8±0.2, very close to values reported in literatures^3,4^.

1. **Dispersion Fitting**

The models we use in determining the phonon dispersion are as follows, with all energy variables in units of eV,

where *E_el_* is the electron energy, *v_F_* is the Fermi velocity, k is the electron wave vector relative to the *K* point, *E_ph_* is the phonon energy, *E_0_* is the phonon energy at the K point, *v_ph_^(1)^* and *v_ph_^(2)^* are the phonon velocities along the *2D_1_* and *2D_2_* directions of the Brillouin zone, and *q_1_* and *q_2_* are the phonon wave vectors relative to the *K* point. Assuming electron-hole symmetry, the phonon wave vector can be solved for, given the measured phonon energy and incident photon energy:

where in the last equation momentum is converted into wavenumber units and we have used a new variable *x*, which is directly measurable since the laser and phonon energies are known.

Since *x* is what we are able to measure in our experiment, we use it to fit the phonon dispersion. When we replace *q* with x in the phonon energy model and convert to wavenumber units we find

This gives us three parameters to fit to our data, *ω_0_*, *v_ph_^(1)^/v_F_^(1)^*, and *v_ph_^(2)^/v_F_^(2)^*, where we divide our measured values into one set for the neutral data and one set for the charged data.

Table S1. Neutral Data (+)

| I | Laser λ(nm) | 2D Peak | *ω_ph_*  (cm^-1^) | *x_1_*×10^-4^ (cm^-1^) | *x_2_*×10^-4^ (cm^-1^) |
| --- | --- | --- | --- | --- | --- |
| 1 | 633 | 1 | 1325 | 1.4473 | 0 |
| 2 | 633 | 2 | 1327 | 0 | 1.4471 |
| 3 | 532 | 1 | 1343 | 1.7454 | 0 |
| 4 | 532 | 2 | 1349 | 0 | 1.7448 |

| I | Laser λ(nm) | 2D Peak | *ω_ph_*  (cm^-1^) | *x_1_*×10^-4^ (cm^-1^) | *x_2_*×10^-4^ (cm^-1^) |
| --- | --- | --- | --- | --- | --- |
| 1 | 633 | 1 | 1325 | 1.4473 | 0 |
| 2 | 633 | 2 | 1327 | 0 | 1.4471 |
| 3 | 532 | 1 | 1342 | 1.7455 | 0 |
| 4 | 532 | 2 | 1345 | 0 | 1.7452 |

Table S2. Charged Data (-)

The best-fit values for *ω_0_*, *v_ph_^(1)^/v_F_^(1)^*, and *v_ph_^(2)^/v_F_^(2)^* are found separately for the neutral and charged data by minimizing the mean squared error of the model,

$$Error=\frac{1}{4}\sum_{I=1}^{4} \left(\omega_{ph,I}-\omega_{0}-\frac{v_{ph}^{(1),(2)}}{v_{F}^{(1),(2)}}x_{1,2,I}\right)^{2}$$

and the best-fit values are listed in Table S3 below.

| Data Set | *ω_0_* (cm^-1^) | *v_ph_^(1)^/v_F_^(1)^* | *v_ph_^(2)^/v_F_^(2)^* |
| --- | --- | --- | --- |
| Neutral | 1229 | 6.5 × 10^-3^ | 6.8 × 10^-3^ |
| Charged | 1238 | 6.0 × 10^-3^ | 6.1 × 10^-3^ |

**Table S3. Best-fit model parameters**

1. **Transport measurement**

We estimate the doping variation of the encapsulated graphene based on the transport measurement data measured on several similar edge-contact encapsulated samples [2]. Fig. S9 shows the estimation measured on a similar sample with hole mobility of 3.2 × 10^4^ cm^2^/Vs at room temperature with formula^2^

$$\sigma=((e\mu\left|n\right|+\sigma_{0})^{-1}+\rho_{s})^{-1}$$

where n is the carrier density value relative to the CNP, *σ_0_* is the residual conductivity at CNP and *ρ_s_* is short range scattering induced resistivity. The residual conductivity, *σ_0_* = 0.36 mS measured from this sample, includes quantum conductance *4e2/h* = 0.15 mS and conductance caused by the charge variation. This gives us an estimation of the upper bound of the effective doping impurity$n_{imp}\leq\frac{\sigma_{0}-\frac{4e^{2}}{h}}{e\mu}=0.4\text{ }\times\text{ }10^{11}cm^{-2}$.


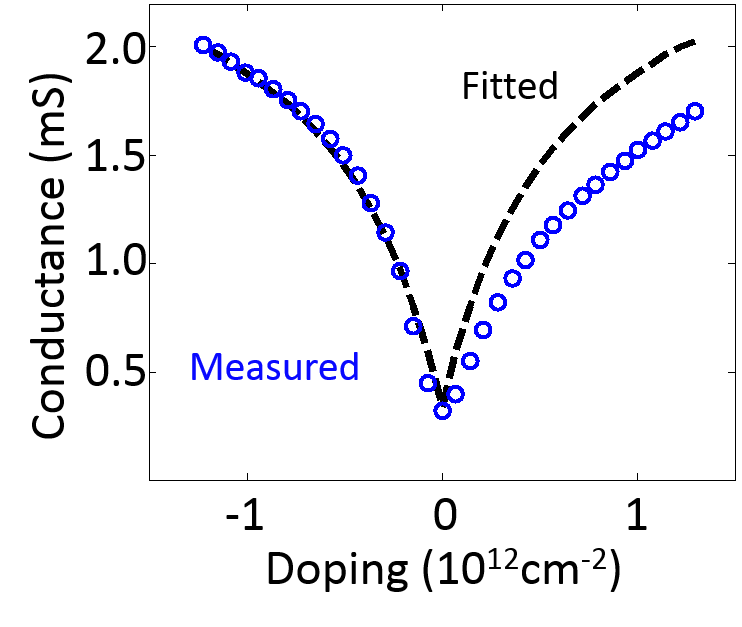


**Figure S9**: Transport data of edge-contact encapsulated graphene (shown in blue). The black dashed line is the fitted data with doping variation not exceeding 0.4 × 10^11^ cm^-2^.

1. **Ratio of Phonon Contour Widths**

There are three pieces of information necessary for computing the ratio of the inner to outer participatory contour widths: 1) FWHM of the 2D_1_ and 2D_2_ peaks, 2) the ratio of the outer to inner Fermi velocities, and 3) phonon to Fermi velocity ratios for the 2D_1_ and 2D_2_ processes.

$$\frac{\delta q_{i}}{\delta q_{o}}=\frac{\delta\omega_{i}}{\delta\omega_{o}}\frac{(v_{ph}/v_{F})_{o}}{(v_{ph}/v_{F})_{i}}\frac{v_{F,o}}{v_{F,i}}$$

where $\delta q$is the contour width, $\delta\omega$ is the FWHM, $v$ is a velocity with either “ph” to denote phonon or “F” to denote Fermi, and inner and outer values denoted by subscript “i”s and “o”s. While we have determined the FWHMs and velocity ratios for our data there is some doping in our sample, so a contour width ratio computed directly from our data cannot be compared with the theoretical value computed assuming zero doping. We address the charge difference between experiment and theory by extrapolating the contour width ratio from our data to the CNP by establishing a linear relationship between 2D peak split and electron density. This linear relationship is prescribed by equating the mean and standard deviation of the 2D peak split with the mean and standard deviation of the electron density as determined by transport measurements.

$$\begin{matrix} \overline{n}=m\overline{\Delta}+b \\ \sigma_{n}=m\sigma_{\Delta} \end{matrix}$$

where n is the electron density, $\Delta$ is the 2D peak split, m is the slope of the linear relationship, b is the y-intercept of the relationship, bars denote means, and $\sigma$s denote standard deviations. The mean and standard deviation of $\Delta$are -8.1 and 3.6 cm^-1^ respectively, and the standard deviation of n is 0.4 × 10^11^ cm^-2^ as discussed in the previous section (doping variation). The mean value of n is 1.4 × 10^11^ cm^-2^, which is determined by using the backgate capacitance of our sample to convert the backgate voltage with minimal conductivity to electron density. These values for the means and standard deviations give a slope of 0.11 × 10^11^ cm^-1^ and y-intercept of 2.32 × 10^11^ cm^-2^ for the relationship between 2D peak split and electron density. This relationship places the CNP at a 2D peak split of 20.4 cm^-1^.

To calculate the outer to inner Fermi velocity ratio we compute the band structure via the well-established tight-binding model for graphene^5^. The inner and outer Fermi velocities are computed as half the 532 nm laser energy divided by the corresponding momentum magnitudes relative to the K point, taken along the K$\Gamma$ and KM directions respectively. These calculations give 1.152 as the ratio of inner to outer Fermi velocities.

Since our data does not directly allow us to calculate the contour width ratio at the CNP, we estimate the ratio at the CNP by assuming a linear relationship between contour width ratio and 2D peak split. In S1g we fit the dispersion to mean values of peak positions where the mean is taken over the appropriate subset of data, “pristine” 2D_1_ excited with 532 nm photons for example. Alternatively in this section we pick an observation of peak positions as excited with the 532 nm laser and pair that observation with each observation of peak positions as excited with the 633 nm laser. For each paring of observations we calculate the best fit dispersion parameters (*ω_0_, v_ph_^(1)^/v_F_^(1)^* , *v_ph_^(2)^/v_F_^(2)^*) from which we calculate the contour width ratio. Figure S10 shows the contour width ratio for each observation excited with the 532 nm laser plot against the 2D peak split of the 532 nm observation. We have additionally averaged the width ratio over the 633 nm observations as otherwise there would be too many data points to visualize well. The blue data and lines assume the 2D_1_ process is the inner process and the 2D_2_ is the outer process, and the black data assumes the reverse. As figure S10 shows there is a strong linear trend between contour width ratio and 2D peak split assuming either correspondence between 2D peak and inner/outer processes. Next we boot strap by resampling with replacement the 533 nm observations and 633 nm observations independently to statistically replicate our experiment another 1000 times. For each statistical replication of the experiment we compute the linear regression between contour width ratio and 2D peak splitting and the mean squared error of the regression. We then find the 99% confidence interval for our contour width ratio extrapolation by computing the contour width ratio using each regression with a mean squared error in the bottom 99% percentile and taking the maximum and minimum values from this set. The confidence intervals are shown in figure S10 with dashed lines. Near the data the confidence interval is tight as expected (there is lots of data), but away from the data points the confidence intervals widen reflecting the diminished certainty of the extrapolation.

Figure S10: Extrapolation of contour width ratio at the CNP using linear regression. The grey region around the CNP reflects the uncertainty we have in determining the CNP. The confidence intervals are at 99% level as determined by boot strapping our data 1000 times.

Putting all these pieces together we find that the ratio of the inner to outer participatory contour widths is equal to 1.3 if the 2D_1_ process corresponds with the *inner* process and the 2D_2_ process corresponds with the *outer* process. But if the 2D_1_ process corresponds with the *outer* process and the 2D_2_ process corresponds with the *inner* process, then the ratio of contour widths is only 0.8. Since this ratio must be greater than one^6^, we conclude that the 2D_1_ process corresponds with the *inner* process and the 2D_2_ process corresponds with the *outer* process. Our conclusion is even robust against significant uncertainty in the CNP. The robustness is shown in figure S10 where the uncertainty in the CNP is shown as a grey region around our best estimate of the CNP. Throughout the entire grey region the contour width ratio is greater than 1 even at the 99% level if the 2D_1_ process corresponds with the *inner* process. This means there is a very low probability, < 0.5%, for a false positive for our claim. Similarly the contour width ratio is less than 1 at the 99% level under the hypothesis that the 2D_1_ process corresponds with the *outer* process, which corresponds with a less than 0.5% probability for falsely rejecting this hypothesis.

**References**

1. Ferrari, A. C. Raman spectroscopy of graphene and graphite: Disorder, electron-phonon coupling, doping and nonadiabatic effects. *Solid State Commun.* **143,** 47–57 (2007).

2. Gannett, W. *et al.* Boron nitride substrates for high mobility chemical vapor deposited graphene. *Appl. Phys. Lett.* **98,** 242105 (2011).

3. Lee, J. E., Ahn, G., Shim, J., Lee, Y. S. & Ryu, S. Optical separation of mechanical strain from charge doping in graphene. *Nat. Commun.* **3,** 1024 (2012).

4. Wang, X., Tantiwanichapan, K., Christopher, J. W., Paiella, R. & Swan, A. K. Uniaxial Strain Redistribution in Corrugated Graphene: Clamping, Sliding, Friction, and 2D Band Splitting. *Nano Lett.* **15,** 5969–5975 (2015).

5. Castro Neto, A. H. *et al.* The electronic properties of grapheme. *Rev. Mod. Phys.* **81,** 109 (2009).

6. Venezuela, P., Lazzeri, M. & Mauri, F. Theory of double-resonant Raman spectra in graphene: Intensity and line shape of defect-induced and two-phonon bands. *Phys. Rev. B* **84,** 35433 (2011).
